# Redox regulation of PDE6 and cGMP signaling in diabetic retinal neurodegeneration

**DOI:** 10.1016/j.bbrep.2026.102670

**Published:** 2026-06-16

**Authors:** Cinthia Jhovanna Perez-Martinez, Lidianys Maria Lewis-Luján, Carlos Alexis Cota-Arrayales, Diego Emmanuel Guerrero-Magaña, Judas Tadeo Vargas-Durazo, Maxim V. Trushin, Mikhail A. Osadchuk, Annette Pulcherie Iloki-Lewis, Juan Carlos Galvez-Ruiz, Simon Bernard Iloki-Assanga

**Affiliations:** a Department of Biological Chemical Sciences. Sonora University, Blvd. Luis Encinas, Col. Centro, Hermosillo, Sonora, C.P. 83000, Mexico; b Department of Medicine and Health Sciences. University of Sonora, Blvd. Luis Encinas, Col. Centro, Hermosillo, Sonora, C.P. 83000, Mexico; c Federal State Autonomous Education Institution of Higher Training, First Sechenov Moscow State Medical University, Moscow, Russian Federation; d Kazan Federal University, Kazan, Russian Federation

**Keywords:** Oxidative stress, Reactive oxygen species (ROS), Phosphodiesterase 6 (PDE6), cGMP signaling, Diabetic retinopathy, Retinal neurodegeneration

## Abstract

Diabetic retinal neurodegeneration is increasingly recognized as an early and critical component of diabetic retinopathy, driven in part by persistent oxidative stress and dysregulated intracellular signaling. Among these pathways, cyclic guanosine monophosphate (cGMP) signaling plays a central role in photoreceptor function and survival. Phosphodiesterase 6 (PDE6), the key enzyme responsible for cGMP hydrolysis in photoreceptors, has been extensively studied in inherited retinal disorders; however, its regulation under diabetic and redox-imbalanced conditions remains insufficiently defined.

In this review, we examine the emerging role of redox imbalance in modulating PDE6 activity and stability in the diabetic retina. We discuss how mitochondrial and non-mitochondrial sources of reactive oxygen species (ROS) may disrupt PDE6 through proteostasis-related mechanisms involving AIPL1-dependent maturation and FAT10-mediated degradation. These alterations may lead to cGMP dysregulation, impaired ion channel activity, calcium imbalance, and photoreceptor dysfunction.

We propose that PDE6-cGMP signaling represents a redox-sensitive hub linking oxidative stress to early neuronal damage in diabetic retinopathy. This mechanistic framework highlights PDE6 as a potential molecular target and supports the development of redox-based strategies aimed at preserving retinal function and preventing neurodegeneration.

## Highlights

• Oxidative stress may destabilize PDE6-dependent phototransduction signaling.
• Proteostasis pathways involving AIPL1 and FAT10 regulate PDE6 integrity.
• CGMP dysregulation contributes to calcium imbalance and neuronal dysfunction.
• PDE6 may represent a mechanistic link between ROS and retinal neurodegeneration.
• Redox-targeted preservation of PDE6 signaling may offer therapeutic potential.

## Graphical abstract

**Figure:**
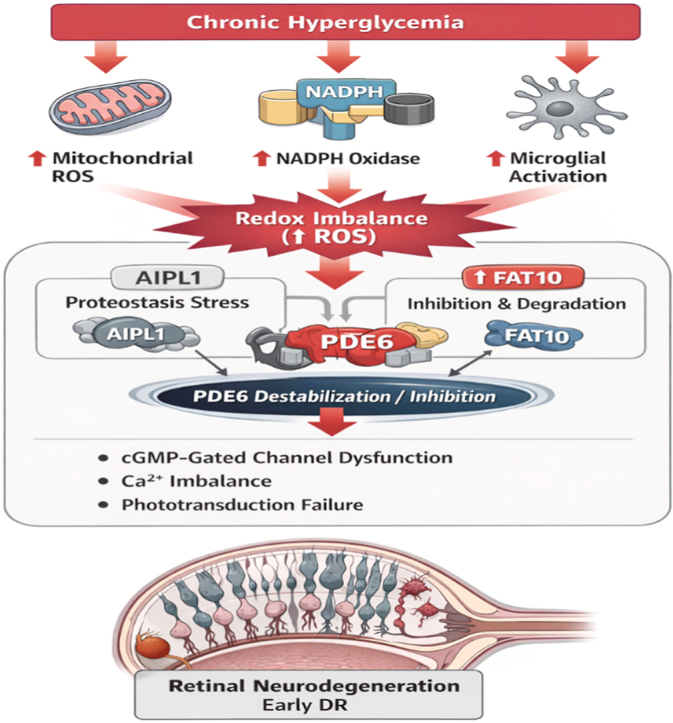

## 1 Introduction

Diabetic retinopathy (DR) remains one of the leading causes of visual impairment worldwide and represents a major complication of diabetes mellitus. Traditionally regarded as a microvascular disorder, growing evidence now supports the concept that DR also involves early neuronal dysfunction and progressive retinal neurodegeneration, which may precede clinically detectable vascular alterations. Photoreceptor cells, retinal ganglion cells, and Müller glia exhibit structural and functional changes during the initial stages of diabetes, indicating that metabolic and molecular stressors contribute significantly to neuronal damage in the diabetic retina [[1], [2], [3]].

Among the molecular mechanisms implicated in DR pathogenesis, oxidative stress has emerged as a central and unifying driver of retinal injury. Chronic hyperglycemia promotes excessive production of reactive oxygen species (ROS) while simultaneously impairing endogenous antioxidant defenses, resulting in persistent redox imbalance within retinal tissues. Both mitochondrial dysfunction and non-mitochondrial sources of ROS, including NADPH oxidases, xanthine oxidase, and activated microglia, have been strongly implicated in the progression of retinal neurodegeneration in diabetes [[4], [5], [6]]. Beyond causing generalized cellular damage, oxidative stress also alters intracellular signaling pathways and protein homeostasis, thereby influencing the stability and function of key photoreceptor proteins.

Photoreceptors are among the most metabolically active cells in the body and operate under conditions of high oxygen consumption and continuous oxidative challenge. Consequently, proteins involved in phototransduction represent particularly vulnerable molecular targets under redox-imbalanced conditions. Phosphodiesterase-6 (PDE6), a central enzyme in the visual transduction cascade, regulates intracellular levels of cyclic guanosine monophosphate (cGMP) in rod and cone photoreceptors. By hydrolyzing cGMP, PDE6 controls the activity of cGMP-gated ion channels and plays a critical role in maintaining photoreceptor membrane polarization and visual signaling [7,8].

Recent studies have demonstrated that the stability, maturation, and functional integrity of PDE6 depend on tightly regulated proteostasis mechanisms involving the specialized chaperone aryl hydrocarbon receptor-interacting protein like 1 (AIPL1). AIPL1 is essential for proper folding and assembly of PDE6, and its dysfunction leads to severe retinal degeneration in inherited retinal disorders [9,10]. In addition, the ubiquitin-like modifier FAT10 has been identified as a regulator of PDE6 activity, promoting its proteasomal degradation and suggesting that protein quality-control pathways critically govern PDE6 stability under stress conditions [11].

**Fig. 1:**
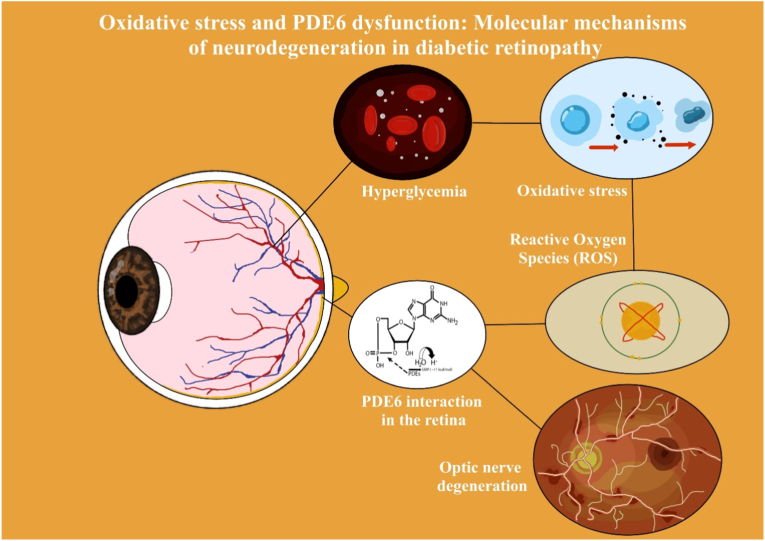
Schematic representation of the molecular mechanisms involved in neurodegeneration in diabetic retinopathy.

Despite these advances, the direct role of PDE6 in diabetic retinal neurodegeneration remains insufficiently characterized. Based on current evidence, oxidative stress may disrupt photoreceptor signaling not only through generalized cellular damage but also by targeting specific molecular components of the phototransduction machinery. In this context, we propose that PDE6 represents a redox-sensitive signaling hub whose dysfunction is driven by proteostasis-dependent mechanisms involving AIPL1 and FAT10. In this review, we examine emerging evidence linking redox imbalance to dysregulation of the PDE6–cGMP signaling axis in the diabetic retina and discuss its implications for photoreceptor dysfunction and early neurodegeneration (Fig. 1).

## 2 Oxidative stress in diabetic retinopathy

Oxidative stress plays a pivotal role in the pathogenesis of diabetic retinopathy and has been recognized as a major driver of retinal neurovascular damage. Persistent hyperglycemia leads to increased generation of reactive oxygen species (ROS) through multiple pathways, including mitochondrial electron transport chain dysfunction, activation of NADPH oxidases, and metabolic flux through the polyol and hexosamine pathways [6,12].

The diabetic retina is particularly susceptible to oxidative damage due to its high metabolic activity and oxygen consumption. Photoreceptors continuously generate ROS as a by-product of mitochondrial oxidative phosphorylation, and hyperglycemic conditions further amplify this oxidative burden. Excessive ROS production disrupts mitochondrial integrity, alters cellular metabolism, and activates inflammatory signaling pathways that promote neuronal degeneration [3,4].

In addition to mitochondrial ROS, several non-mitochondrial sources contribute to oxidative stress in the diabetic retina. NADPH oxidase isoforms expressed in retinal endothelial cells, Müller glia, and microglia generate superoxide in response to hyperglycemia and inflammatory stimuli. Activation of microglia further amplifies ROS production and promotes neuroinflammatory responses that exacerbate retinal damage [5,13].

Importantly, oxidative stress not only damages cellular structures but also modulates signaling pathways that regulate protein stability, enzymatic activity, and gene expression. These effects may have profound consequences for photoreceptor signaling proteins, including PDE6, which plays a central role in maintaining cGMP homeostasis in the retina.

## 3 Proteostasis regulation of PDE6

PDE6 is a critical enzyme in the phototransduction cascade and is responsible for the hydrolysis of cyclic guanosine monophosphate (cGMP) in rod and cone photoreceptors. Tight regulation of PDE6 activity is essential for maintaining photoreceptor sensitivity and visual signal transduction [7].

The maturation and stability of PDE6 depend on the specialized chaperone AIPL1, which facilitates proper folding and assembly of the enzyme complex. Loss of AIPL1 function disrupts PDE6 biogenesis and leads to rapid photoreceptor degeneration, highlighting the importance of proteostasis mechanisms in maintaining photoreceptor function [9,10].

Recent studies have also identified FAT10 as a regulator of PDE6 stability. FAT10 is a ubiquitin-like modifier involved in targeting proteins for proteasomal degradation. Boehm et al. (2020) demonstrated that FAT10 can interact with PDE6 and inhibit its enzymatic activity while promoting proteasomal degradation of the enzyme. These findings suggest that inflammatory and stress-related signaling pathways may modulate PDE6 abundance through FAT10-dependent mechanisms.

Under conditions of oxidative stress, proteostasis networks become increasingly challenged. Accumulation of misfolded proteins, impaired chaperone activity, and activation of proteasomal degradation pathways may destabilize critical signaling proteins such as PDE6. Therefore, chronic oxidative stress in the diabetic retina may disrupt the AIPL1–FAT10–PDE6 regulatory axis, ultimately leading to dysregulation of cGMP signaling and photoreceptor dysfunction.

### 3.1 Phosphodiesterase 6 and cGMP

Phosphodiesterase 6 (PDE6) is the principal cGMP-hydrolyzing enzyme in rod and cone photoreceptors and plays a critical role in maintaining retinal signal transduction and photoreceptor homeostasis [7,8,[14], [15], [16], [17], [18]]. Tight regulation of intracellular cGMP levels is essential because disturbances in cGMP metabolism can alter cGMP-gated channel activity, disrupt calcium homeostasis, and promote photoreceptor dysfunction [7,8,[17], [18], [19]].

**Fig. 2:**
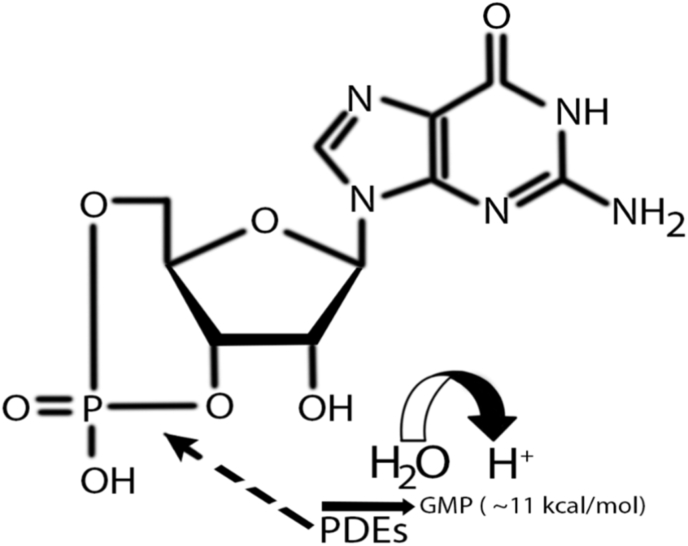
Schematic illustration of the PDE reaction of cGMP, showing the 3′ cyclic phosphate bond of cGMP hydrolyzed by PDEs.

Beyond its physiological role in phototransduction, growing evidence suggests that dysregulation of PDE6-dependent cGMP signaling may contribute to retinal neurodegeneration (Fig. 2).

## 4 PDE6–cGMP signaling as a redox vulnerable hub in the diabetic retina

PDE6 is the principal cGMP phosphodiesterase in rod and cone photoreceptors and a core determinant of phototransduction gain and temporal dynamics. Upon light activation, the rhodopsin transducing cascade relieves PDE6 inhibition, accelerating cGMP hydrolysis, closing cGMP-gated channels, and driving photoreceptor hyperpolarization. [8,9] Given the steep functional dependence of photoreceptor physiology on cGMP dynamics, modest perturbations in PDE6 activity or abundance can translate into major disruptions of ionic balance, calcium homeostasis, and synaptic signaling [9,19].

### 4.1 Oxidative stress in DR: convergence onto photoreceptor proteostasis and signaling

Oxidative stress in DR is now supported by extensive mechanistic and translational evidence. Recent comprehensive reviews emphasize that sustained ROS contributes not only to vascular pathology but also to retinal neurodegeneration, inflammation, and metabolic reprogramming, with mitochondrial and NADPH oxidase pathways playing prominent roles.

Although PDE6 represents the principal photoreceptor phosphodiesterase, other phosphodiesterase isoforms may also contribute to diabetic retinal dysfunction. PDE5, PDE9, and other cGMP-regulating phosphodiesterases participate in vascular tone regulation, inflammatory signaling, and endothelial homeostasis, suggesting that diabetic retinal injury likely involves broader cyclic nucleotide dysregulation beyond photoreceptors alone. Furthermore, retinal neurodegeneration in diabetic retinopathy occurs in parallel with classical vascular abnormalities including endothelial dysfunction, capillary dropout, blood-retinal barrier disruption, and neuroinflammation. Integrating photoreceptor dysfunction with these neurovascular alterations may provide a more comprehensive understanding of disease progression and translational therapeutic opportunities. This environment is especially relevant to photoreceptors, which operate at high oxygen consumption and are susceptible to redox-driven damage and maladaptive stress responses [7,8,[16], [17], [18]].

### 4.2 AIPL1-dependent maturation of PDE6: a proteostasis checkpoint sensitive to stress

Functional PDE6 requires correct folding, assembly, and post-translational maturation. AIPL1 has been established as an obligate chaperone of PDE6, enabling productive folding and stabilizing nascent PDE6 in conjunction with partner chaperone systems [11,20]. Structural and mechanistic studies have further clarified that AIPL1 contains specialized domains that bind prenylated PDE6 subunits and interface with broader chaperone machinery, positioning AIPL1 as a critical proteostasis checkpoint for PDE6 biogenesis [10,21].

From a redox perspective, chronic oxidative stress may compromise this proteostasis checkpoint by shifting cellular resources toward stress adaptation, altering chaperone–client interactions, and promoting degradation-prone PDE6 intermediates. However, direct oxidative modification of PDE6 or disruption of the AIPL1–PDE6 complex has not yet been experimentally demonstrated in diabetic retinal tissue. Therefore, the proposed involvement of AIPL1 dysfunction in diabetic retinal neurodegeneration should currently be considered a plausible mechanistic hypothesis requiring experimental validation, although available evidence supports the biological plausibility of this mechanism and warrants further investigation [11,20,22].

### 4.3 FAT10 as an inhibitory and degradative regulator of PDE6: a mechanistic bridge between stress/inflammation and cGMP dysregulation

A key mechanistic advance relevant to this hypothesis is the demonstration that FAT10 targets PDE6 for proteasomal degradation via covalent conjugation and can also inhibit PDE6 enzymatic activity through non-covalent interactions with regulatory/catalytic domains [1]. Importantly, this work also showed FAT10 expression in human retina and identified PDE6 as a retina specific substrate, providing biological plausibility for FAT10 driven PDE6 loss in disease contexts characterized by inflammation and cellular stress [23]. Importantly, FAT10-mediated degradation of PDE6 has been experimentally demonstrated in retinal systems. Nevertheless, direct evidence linking FAT10-dependent PDE6 degradation to diabetic retinal neurodegeneration remains unavailable. Consequently, the extrapolation of this mechanism to diabetic retinopathy should be interpreted cautiously until validated in diabetes-specific retinal models.

Because FAT10 pathways are closely linked to stress and inflammatory signaling, oxidative stress in DR may plausibly amplify FAT10-mediated pressure on PDE6 abundance and activity, producing sustained cGMP dysregulation. Such dysregulation can alter cGMP-gated channel behavior and downstream calcium influx, ultimately promoting photoreceptor dysfunction and neurodegenerative signaling cascades [7,8,11,17,18].

Hypothetically, chronic oxidative stress may initiate a sequential disruption of PDE6 proteostasis and signaling integrity. Although this model is biologically plausible and supported indirectly by evidence from oxidative stress and inherited retinal degeneration studies, several proposed molecular events remain experimentally unverified in diabetic retinal tissue. Excessive ROS production in diabetic photoreceptors can induce oxidative modification of susceptible amino acid residues, including cysteine oxidation and S-nitrosylation within PDE6 catalytic and regulatory domains. These structural alterations may destabilize interactions between catalytic PDE6 subunits and inhibitory Pγ subunits, thereby compromising enzymatic stability and activity. Concurrently, oxidative stress may impair AIPL1-dependent chaperone activity, reducing the efficiency of PDE6 folding and maturation while increasing the accumulation of unstable intermediates. Under sustained inflammatory and stress conditions, FAT10-dependent proteasomal pathways may further enhance PDE6 degradation, collectively leading to reduced cGMP hydrolysis, persistent cGMP accumulation, dysregulation of cGMP-gated ion channels, calcium overload, and progressive photoreceptor dysfunction.

These observations further support the hypothesis that oxidative stress may destabilize PDE6 proteostasis and signaling integrity in diabetic retinal neurodegeneration.

### 4.4 Working model: redox-driven PDE6 destabilization → cGMP imbalance → neurodegeneration

Collectively, the available evidence supports a working hypothesis in which mitochondrial and non-mitochondrial ROS sources may converge on photoreceptor proteostasis and signaling pathways, potentially destabilizing PDE6 through AIPL1-dependent quality-control mechanisms and FAT10-mediated degradation. However, several components of this proposed redox–AIPL1–FAT10–PDE6 axis remain to be experimentally validated in diabetic retinal models [1,2,4,5,[7], [8], [9],^17^,^18^,^21^,^24^,^25^]. This redox proteostasis signaling intersection may represent a tractable mechanistic link between oxidative stress and early neuronal dysfunction in DR.

Importantly, although substantial evidence supports the involvement of oxidative stress, photoreceptor dysfunction, and cGMP dysregulation in diabetic retinopathy, direct experimental demonstration that PDE6 acts as a primary redox-sensitive hub in diabetic retinal neurodegeneration remains limited [[1], [2], [3], [4], [5], [6],[23], [24], [25], [26]].

### 4.5 Current limitations and knowledge gaps

Although the proposed PDE6-centered redox model provides a coherent mechanistic framework, it should currently be regarded as a hypothesis-generating model rather than an experimentally established pathogenic pathway in diabetic retinopathy.

Despite growing interest in photoreceptor dysfunction during diabetic retinal neurodegeneration, several important limitations currently restrict mechanistic interpretation of the PDE6-centered redox model. First, direct evidence demonstrating oxidative modification or destabilization of PDE6 in diabetic retinal tissue remains scarce. Most available data derive from inherited retinal degeneration models or generalized oxidative stress paradigms rather than diabetes-specific experimental systems [[3], [4], [5],[9], [10], [11]].

Second, the temporal relationship between oxidative stress, proteostasis disruption, and PDE6 dysfunction remains unresolved. Whether PDE6 dysregulation represents an initiating event or a secondary consequence of broader retinal metabolic failure is currently unknown [[4], [5], [6],^25^,^26^].

Third, the molecular mechanisms linking ROS-mediated stress to AIPL1 dysfunction and FAT10 activation in diabetic photoreceptors have not yet been experimentally defined. Similarly, the contribution of post-translational oxidative modifications, including cysteine oxidation or S-nitrosylation of PDE6 subunits, requires direct biochemical investigation [[8], [9], [10], [11],^17^,^21^].

Furthermore, the extent to which oxidative stress **may contribute** to AIPL1 dysfunction or FAT10 activation in diabetic photoreceptors is currently unknown. Finally, diabetic retinopathy is a complex neurovascular disease involving endothelial dysfunction, inflammation, gliosis, mitochondrial impairment, and neuronal degeneration. Therefore, PDE6 dysfunction likely represents only one component within a broader network of pathogenic mechanisms. Future studies using diabetic animal models, human retinal tissue, and high-resolution proteomic approaches will be essential to validate the translational relevance of this proposed signaling axis [[1], [2], [3], [4], [5], [6],[23], [24], [25], [26]].

### 4.6 Translational and therapeutic perspectives

Nevertheless, direct therapeutic targeting of PDE6 presents several translational challenges. Because PDE6 is essential for normal phototransduction, excessive modulation of its activity could potentially impair visual signaling and retinal adaptation [7,8,[14], [15], [16], [17], [18]]. In addition, selective delivery of PDE6-targeted therapies to photoreceptors remains technically challenging in vivo due to retinal compartmentalization and blood-retinal barrier limitations [[1], [2], [3], [4], [5], [6],^25^,^26^]. Consequently, future therapeutic approaches may benefit from indirectly preserving PDE6 function through redox modulation, mitochondrial protection, proteostasis stabilization, or anti-inflammatory interventions rather than direct enzymatic inhibition or activation [4,5,[9], [10], [11],^25^,^26^].

Such approaches **may plausibly regulate** upstream pathogenic processes while avoiding direct interference with physiological phototransduction signaling (Table 1).

**Table 1:** summarizes the principal oxidative stress sources, PDE6 regulatory mechanisms, downstream signaling consequences, and potential therapeutic approaches implicated in diabetic retinal neurodegeneration.

ROS/Stress Source	PDE6-Related Mechanism	Downstream Consequence	Potential Therapeutic Strategy	References
Mitochondrial ROS	Oxidative destabilization of PDE6 and disruption of photoreceptor proteostasis	cGMP accumulation, calcium dysregulation, and photoreceptor dysfunction	Mitochondrial antioxidants and redox-modulating therapies	[[4], [5], [6],^13^,^25^,^26^]
NADPH oxidase activation	Increased oxidative burden on retinal neurons and photoreceptors	Neuroinflammation and signaling disruption	NADPH oxidase inhibitors and anti-inflammatory therapies	[5,6,12,13,23,25,26]
AIPL1 dysfunction	Impaired PDE6 folding, assembly, and maturation	Phototransduction instability and reduced PDE6 activity	Proteostasis stabilization and chaperone-targeted approaches	[9,10,20,21]
FAT10 activation	Proteasomal degradation and inhibition of PDE6	Reduced cGMP hydrolysis and signaling imbalance	Anti-inflammatory modulation and proteostasis-preserving interventions	[7,8,11,17,18]
Chronic hyperglycemia	Persistent oxidative stress and redox imbalance affecting retinal signaling pathways	Retinal neurodegeneration and neuronal dysfunction	Redox-targeted neuroprotection and metabolic control strategies	[[1], [2], [3], [4], [5], [6],^12^,^13^,[24], [25], [26]]

## 5 Conclusions and future perspectives

Diabetic retinopathy is increasingly recognized as a neurovascular and neurodegenerative disease in which chronic redox imbalance acts as a central driver of retinal dysfunction. Contemporary evidence highlights that oxidative stress contributes to neuronal injury, inflammatory amplification, and progressive retinal degeneration, arising from both mitochondrial dysfunction and non-mitochondrial sources of reactive oxygen species (ROS) such as NADPH oxidases and activated innate immune cells [4,5,7,25,26].

In this context, we propose that PDE6 **may represent** a redox-sensitive signaling hub linking oxidative stress to cGMP dysregulation and photoreceptor dysfunction. Based on currently available evidence, we propose that oxidative stress may influence PDE6 stability and activity through proteostasis-dependent mechanisms involving AIPL1-mediated maturation and FAT10-mediated degradation. Nevertheless, direct experimental validation of this proposed signaling axis in diabetic retinal tissue remains necessary [[9], [10], [11],^21^]. In this framework, sustained PDE6 dysfunction may promote cGMP imbalance, impair phototransduction fidelity, perturb ionic and calcium homeostasis, and contribute to early retinal neurodegeneration.

This integrative model provides a mechanistic basis connecting mitochondrial dysfunction, ROS overproduction, and neuronal degeneration in the diabetic retina. Future studies should focus on: (i) identifying redox-dependent modifications regulating PDE6 biogenesis, stability, and activity; (ii) defining the temporal dynamics between ROS generation, AIPL1/FAT10 pathway modulation, and PDE6 dysfunction; and (iii) evaluating whether redox-targeted interventions can preserve PDE6 function and retinal integrity [4,5,11,25,26]. Particular emphasis should be placed on determining whether the proposed redox–AIPL1–FAT10–PDE6 axis exists in diabetic retinal tissue, as this mechanism **remains to be experimentally validated**.

Collectively, these findings support the concept that PDE6 **may represent** a promising molecular target for mechanism-based neuroprotective strategies in diabetic retinopathy. Nevertheless, the proposed signaling axis **should be considered a mechanistic hypothesis** and **requires validation in diabetic retinal models** before definitive therapeutic conclusions can be drawn.

## Consent for publication

All authors have given permission to submit the paper for publication.

## Ethics approval statement

This is a review manuscript, and no human or animal data was used. Ethical approval is therefore not needed for the study.

## Funding statement

No funding was obtained for this study.

## CRediT authorship contribution statement

**Cinthia Jhovanna Perez-Martinez:** Conceptualization, Data curation, Formal analysis, Investigation, Methodology, Writing – original draft, Writing – review & editing. **Lidianys Maria Lewis-Luján:** Conceptualization, Data curation, Formal analysis, Investigation, Methodology, Project administration, Resources, Software, Supervision, Validation, Visualization, Writing – original draft, Writing – review & editing. **Carlos Alexis Cota-Arrayales:** Conceptualization, Data curation, Formal analysis, Investigation, Methodology, Resources, Visualization, Writing – original draft, Writing – review & editing. **Diego Emmanuel Guerrero-Magaña:** Conceptualization, Data curation, Formal analysis, Investigation, Methodology, Resources, Writing – original draft. **Judas Tadeo Vargas-Durazo:** Conceptualization, Data curation, Investigation, Methodology, Resources, Writing – original draft. **Maxim V. Trushin:** Conceptualization, Data curation, Formal analysis, Investigation, Methodology, Project administration, Supervision, Writing – original draft, Writing – review & editing. **Mikhail A. Osadchuk:** Conceptualization, Data curation, Formal analysis, Investigation, Methodology, Writing – original draft, Writing – review & editing. **Annette Pulcherie Iloki-Lewis:** Conceptualization, Data curation, Formal analysis, Investigation, Methodology, Project administration, Software, Supervision, Validation, Visualization, Writing – original draft, Writing – review & editing. **Juan Carlos Galvez-Ruiz:** Conceptualization, Data curation, Formal analysis, Investigation, Methodology, Project administration, Writing – original draft. **Simon Bernard Iloki-Assanga:** Conceptualization, Data curation, Formal analysis, Funding acquisition, Investigation, Methodology, Project administration, Resources, Software, Supervision, Validation, Visualization, Writing – original draft, Writing – review & editing.

## Declaration of competing interest

I hereby confirm no potential competing financial or non-financial interests on behalf of all authors of the manuscript.

## Data Availability

No data was used for the research described in the article.
